# Phthalate metabolites and sex steroid hormones in relation to obesity in US adults: NHANES 2013-2016

**DOI:** 10.3389/fendo.2024.1340664

**Published:** 2024-03-08

**Authors:** Jiechang Zhang, Wen Gu, Shilei Zhai, Yumeng Liu, Chengcheng Yang, Lishun Xiao, Ding Chen

**Affiliations:** ^1^ School of Medical Information and Engineering, Xuzhou Medical University, Xuzhou, Jiangsu, China; ^2^ Department of Biostatistics, School of Public Health, Xuzhou Medical University, Xuzhou, Jiangsu, China; ^3^ Department of Cardiology, Zhuhai People's Hospital (Zhuhai Clinical Medical College of Jinan University), Zhuhai, Guangdong, China; ^4^ Department of Ophthalmology, The Fifth Affiliated Hospital, Sun Yat-Sen University, Zhuhai, Guangdong, China; ^5^ Center for Medical Statistics and Data Analysis, Xuzhou Medical University, Xuzhou, Jiangsu, China; ^6^ Key Laboratory of Human Genetics and Environmental Medicine, Xuzhou Medical University, Xuzhou, Jiangsu, China

**Keywords:** obesity, phthalates, sex hormones, machine learning, NHANES

## Abstract

**Background:**

Obesity and metabolic syndrome pose significant health challenges in the United States (US), with connections to disruptions in sex hormone regulation. The increasing prevalence of obesity and metabolic syndrome might be associated with exposure to phthalates (PAEs). Further exploration of the impact of PAEs on obesity is crucial, particularly from a sex hormone perspective.

**Methods:**

A total of 7780 adult participants in the National Health and Nutrition Examination Survey (NHANES) from 2013 to 2016 were included in the study. Principal component analysis (PCA) coupled with multinomial logistic regression was employed to elucidate the association between urinary PAEs metabolite concentrations and the likelihood of obesity. Weighted quartiles sum (WQS) regression was utilized to consolidate the impact of mixed PAEs exposure on sex hormone levels (total testosterone (TT), estradiol and sex hormone-binding globulin (SHBG)). We also delved into machine learning models to accurately discern obesity status and identify the key variables contributing most to these models.

**Results:**

Principal Component 1 (PC1), characterized by mono(2-ethyl-5-carboxypentyl) phthalate (MECPP), mono(2-ethyl-5-hydroxyhexyl) phthalate (MEHHP), and mono(2-ethyl-5-oxohexyl) phthalate (MEOHP) as major contributors, exhibited a negative association with obesity. Conversely, PC2, with monocarboxyononyl phthalate (MCNP), monocarboxyoctyl phthalate (MCOP), and mono(3-carboxypropyl) phthalate (MCPP) as major contributors, showed a positive association with obesity. Mixed exposure to PAEs was associated with decreased TT levels and increased estradiol and SHBG. During the exploration of the interrelations among obesity, sex hormones, and PAEs, models based on Random Forest (RF) and eXtreme Gradient Boosting (XGBoost) algorithms demonstrated the best classification efficacy. In both models, sex hormones exhibited the highest variable importance, and certain phthalate metabolites made significant contributions to the model’s performance.

**Conclusions:**

Individuals with obesity exhibit lower levels of TT and SHBG, accompanied by elevated estradiol levels. Exposure to PAEs disrupts sex hormone levels, contributing to an increased risk of obesity in US adults. In the exploration of the interrelationships among these three factors, the RF and XGBoost algorithm models demonstrated superior performance, with sex hormones displaying higher variable importance.

## 1 Introduction

The prevalence of central obesity, defined as body mass index (BMI) ≥ 30.0 kg/m^2^, significantly increased from 45.2% in 1999-2000 to 56.7% in 2013-2014 (1). Obese people are also more likely to develop metabolic diseases that threaten population health, such as cardiovascular disease, type 2 diabetes, dyslipidaemia, osteoarthritis, sleep apnoea, certain types of cancer and all-cause mortality (2–4). The increase in obesity rates in the population can be attributed to alterations in genetic, lifestyle, and environmental factors and their interactions (5). Research have proven a tight correlation between sex steroid hormones and obesity. Testosterone (TT) is the major androgenic steroid hormone in adult males and is responsible for maintaining sperm production, libido, and sexual efficacy (6). Men with obesity exhibit reduced levels of testosterone, and sex hormone-binding globulin (SHBG) (7, 8). Obesity-associated reduction in testosterone is accompanied by reduced levels of luteinizing hormone (LH), whereas age-related reduction in testosterone is correlated with increased LH (7), indicating central rather than gonadal dysregulation in obesity. Estradiol, the principal hormone in female reproduction, is vital for the development and maintenance of female reproductive tissues and the regulation of the menstrual cycle (9). Women with overweight and obesity tend to have higher estrogen levels compared to their normal-weight counterparts (10). Weight loss interventions have been shown to effectively reduce estrogen levels among females with obesity (11). SHBG is a glycoprotein that transports TT and estradiol to target tissues, thereby influencing the bioavailability of these reproductive hormones (12). Observational studies have indicated that lower levels of SHBG are associated with an increased incidence of insulin resistance and type 2 diabetes, independent of sex hormone concentrations (13). Sex steroid hormone drugs have been used to treat obesity and metabolic imbalances (14).

Endocrine disruptors chemicals (EDCs) are a group of substances with endocrine hormone effects, most of which are artificially synthesized chemicals, such as bisphenol A, phthalates (PAEs), insecticides, polychlorinated biphenyls, and more (15). These substances can enter the human body through ingestion in the digestive tract, inhalation in the respiratory tract, and skin contact, resulting in a variety of adverse effects, which are mainly characterized by endocrine disruption, hormone function disruption, and reproductive organ developmental disorders, and in severe cases, can induce cancer (16, 17). Evidence suggests that EDCs may be associated with a significant increase in the prevalence of metabolic diseases such as obesity (18). PAEs as EDCs continue to receive academic attention as risk factors for metabolic diseases such as diabetes mellitus, hypertension, hyperlipidemia, and the reproductive toxicity (19, 20). PAEs are mainly used as plasticizers in the manufacturing of plastic products. Plastic products can be found everywhere in modern life, from infants to the elderly, all of whom are exposed to PAEs in the environment for long periods of time. The variety of PAEs used as plasticizers is large, and their hydrolysis process in the human body is complex, with different stages of metabolites (21). Previous studies have indicated the presence of 22 phthalate metabolites in human urine (22). Exposure to PAEs may induce hypothalamic-pituitary-gonadal (HPG) axis dysfunction, disrupting the balance of multiple sex hormones within the body (23, 24). Exposure to PAEs is closely linked to obesity, except for their role in causing imbalances in sex hormone levels. The association between PAEs and obesity has been extensively investigated in diverse populations (25–28). PAEs metabolites exhibit biochemical activity, including the activation of peroxisome proliferator receptors and antiandrogenic effects, which contribute to the development of obesity (29).

Considering the correlation between sex steroid hormones and obesity, we included all these three aspects in our study. To explore the role that sex steroid hormones play in the increased risk of obesity due to phthalates, we tried various machine learning models for interpretation. We aimed to investigate the association between PAEs, sex steroid hormones, and obesity from a novel perspective, thereby highlighting the health risks associated with PAEs.

## 2 Methods

### 2.1 Study design and participants

**Figure 1:**
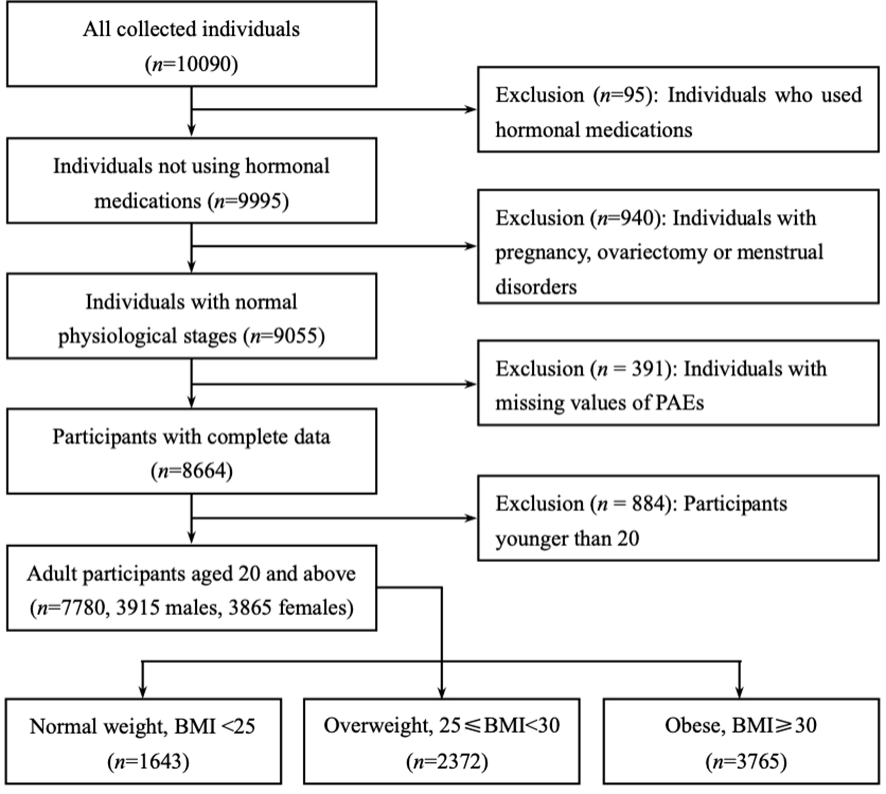
Flow chart of the participants included in this analysis (*n* = 7780), NHANES 2013-16. Only adult participants aged 20 years or older were retained (3915 males and 3865 females). All participants were divided into three subgroups based on body mass index (BMI): BMI of less than 25 were ‘Normal weight’, between 25 and 30 were ‘Overweight’, greater than or equal to 30 were defined as ‘Obese’.

NHANES is an ongoing cross-sectional survey conducted by the National Center for Health Statistics (NCHS) of the Centers for Disease Control and Prevention (CDC) to collect health screening data from a nationally representative sample of U.S. residents and noninstitutionalized civilians. The dataset for this study contains two cycles of NHANES (2013-2014 and 2015-2016), which includes laboratory data on phthalate metabolites and sex steroid hormones (TT, estradiol and SHBG). We initially selected 10090 participants. Of these, 1035 participants were excluded due to the presence of interfering sex hormone levels (including hormone medication using, pregnancy, ovariectomy and menstrual disorders), and a further 391 participants were excluded due to missing data on phthalate variables, resulting in the inclusion of 7780 eligible adult subjects (3915 males and 3865 females). The flow chart for screening participants is shown in **Figure 1**.

### 2.2 Phthalate metabolites

Phthalate metabolites were sampled using urine, and quantitative detection was achieved by high performance liquid chromatography-electrospray ionization-tandem mass spectrometry (30). These metabolites encompass various compounds, including monocarboxynonyl phthalate (MCNP) from di-isodecyl phthalate (DDP), mono(2-ethyl-5-carboxypentyl) phthalate (MECPP), mono(2-ethylhexyl) phthalate (MEHP), mono(2-ethyl-5-hydroxyhexyl) phthalate (MEHHP), and mono(2-ethyl-5-oxohexyl) phthalate (MEOHP) derived from di(2-ethylhexyl) phthalate (DEHP) monocarboxyoctyl phthalate (MCOP) and monoisononyl phthalate (MNP) from di-isononyl phthalate (DNP); mono(3-carboxypropyl) phthalate (MCPP) from di-n-octyl phthalate (DiNOP); mono-n-butyl phthalate (MBP) from di-n-butyl phthalate (DBP); monoethyl phthalate (MEP) from di-ethyl phthalate (DEP); mono-isobutyl phthalate (MiBP) from di-isobutyl phthalate; monobenzyl phthalate (MBzP) from benzylbutyl phthalate (BzBP); and cyclohexane-1,2-dicarboxylic acid-mono(hydroxy-isononyl) ester (MHNCH) from 1,2-cyclohexane dicarboxylic acid, di-isononyl ester (DINCH) (31). We calculated the non-detection rate of individual phthalate metabolites in the samples and excluded two variables with non-detection rates greater than 50%. 11 phthalate metabolites were finally included in this study, namely MCNP, MCOP, MECPP, MBP, MCPP, MEP, MEHHP, MEHP, MiBP, MEOHP and MBzP. The lower limit of detections (LLODs) of all phthalate metabolites are shown in **Table S4**. Detailed protocols for urine sample collection and its analysis are described in publications and on the CDC Web site (32, 33).

### 2.3 Sex steroid hormones

Sex steroid hormone data were categorized in the NHANES laboratory data. Briefly, total testosterone and estradiol were determined using isotope dilution liquid chromatography-tandem mass spectrometry (ID-LC-MS/MS). SHBG is not measured directly, but is based on a chemiluminescent measurement of the reaction of SHBG with an immunological antibody and the reaction products. For detailed descriptions of all laboratory test methods, refer to the CDC’s Laboratory Methods document (34, 35). Sex steroid hormones were measured using blood samples.

### 2.4 Covariates

Several covariates were selected for inclusion in the statistical model based on the characteristics of the population studied. These included some typical demographic variables such as age, gender, race, place of birth, and marital status. Educational level was divided into four categories: lower than high school, high school, some college, or Associate of Arts (AA) degree, and college graduate or above. The ratio of household income to poverty was set to three categories (1.3 and 3.5 as the two dividing lines for the ratio values). Smoking, drinking, hypertension, and diabetes were included in the study as basic diseases of the population. Other key covariates were body mass index (BMI), time of day of serum collection (i.e. morning, afternoon, evening) and urinary creatinine. Considering the possible influence on individual obesity status, we included physical activity and average daily calorie intake as covariates (36).

### 2.5 Statistical analysis

NHANES uses a complex sampling weight design (37), intended to make the sample data more representative of the entire U.S. population. Therefore, we conducted statistical descriptions on the sample data and weighted data separately. The population was divided into three subgroups based on body mass index (BMI): BMI of less than 25 were ‘Normal weight’, between 25 and 30 were ‘Overweight’, greater than or equal to 30 were defined as ‘Obese’. PAEs metabolite levels were derived from urine samples, to control for the interference of renal metabolic differences in different individuals in the study, urinary creatinine levels were adjusted to accommodate variations in urine dilution (36, 38, 39), and the timing of blood sample collection was regulated to address diurnal fluctuations in sex hormone concentrations (36). For continuous variables, skewness and kurtosis tests were used to test whether the distribution of the data was approximately normal, and non-normal continuous variables were described using the median (IQR). The Mann-Whitney U test and Kruskal-Wallis’ rank sum test were used to compare differences between subgroups. For categorical variables, the frequency and percentage were calculated, and the Chi-square test was used to check differences between subgroups. *P* values for multiple comparisons were calibrated using the Bonferroni’s correction.

We plotted correlation heat maps and calculated Spearman’s correlation coefficients and FDR-corrected (false discovery rate) *P* values to confirm the correlation between urinary creatinine and phthalate metabolites. Due to the presence of multicollinearity among the phthalate metabolites, principal component analysis was used to obtain principal component variables for the phthalate metabolite variables, which were included in the multinomial logistic regression instead of the original variables. Weighted quantile sum (WQS) regression was used to explore the effects of mixed PAEs exposure on sex steroid hormones and BMI.

We experimented with a variety of machine learning models to build predictive models with the aim of making full use of data features to shed light on the impact of phthalates on human health. We tested K-Nearest Neighbor (KNN), Naive Bayes, Support Vector Machines (SVM), Decision Trees (DT), Random Forest (RF), Gradient Boosting Decision Tree (GBDT) and eXtreme Gradient Boosting (XGBoost) algorithms to build the predictive models, using cross-validation and calculating the predictive accuracy of each model. ‘Accuracy’ and ‘F1-score’ was calculated to evaluate the superiority of prediction performance among the models.

Data analysis and machine learning modelling were implemented using R of version 4.2.1, with missing values of the independent variables filled in by the “DMwR2” package. Outliers were identified as values outside the interquartile plus or minus three interquartile range (IQR) and were removed from further analysis. Principal component analysis, multinomial logistic regression and WQS regression were implemented by the package “stats”, “nnet” and “gWQS”, respectively. The statistical significance level was set at 0.05. The modelling was mainly performed using a number of machine learning algorithms integrated in the “mlr” package (40).

## 3 Results

### 3.1 Characteristics of subjects

**Table 1** shows the descriptive statistics of the 7780 subjects included in the analysis, which were divided into three groups based on BMI; i.e., 1643 participants were ‘Normal weight’, 2372 participants were ‘overweight’, and 3765 participants were defined as ‘obese’. The median ages for the total group and the three subgroups were 60, 58, 62, and 59, respectively. Non-Hispanic black people were in the majority in each subgroup, followed by non-Hispanic white people. The highest proportion of males was found in the overweight subgroup. Overall, the ‘Overweight’ and ‘Obese’ subgroups had lower educational attainment than the ‘Normal weight’ subgroup. The ‘Obese’ subgroup had a smaller proportion of higher incomes. Over half of the participants in the overweight and obese subgroups were married. The ‘Obese’ subgroup had a higher percentage of US births than overall. The ‘Normal weight’ subgroup had a higher proportion of never-smokers than overall. Notably, the ‘Obese’ subgroup had a higher rate of diabetes, while the ‘Overweight’ subgroup had a higher average daily energy intake. The weighted statistical descriptions of the participants can be viewed in **Table S1**.

**Table 1:** Demographic characteristics in different subgroups.

	Overall	Normal weight	Overweight	Obese	*P* values
*n* (%)	7780 (100)	1643 (21.12)	2372 (30.49)	3765 (48.39)	
Age(years); median (IQR)	60 (25)	58 (34)	62 (24) ^a***^	59 (22) ^b***^	< 0.001
Gender(male), *n* (%)	3915 (50.32)	726 (44.19)	1452 (61.21)	1737 (46.14)	< 0.001
Race/ethnicity, *n* (%)					< 0.001
Mexican American	1091 (14.02)	138 (8.40)	337 (14.21)	616 (16.36)	
Non-Hispanic White People	1786 (22.96)	290 (17.65)	487 (20.53)	1009 (26.80)	
Non-Hispanic Black People	3120 (40.10)	716 (43.58)	927 (39.08)	1477 (39.23)	
Other Hispanic	814 (10.46)	125 (7.61)	295 (12.44)	394 (10.46)	
Other Race	969 (12.46)	374 (22.76)	326 (13.74)	269 (7.14)	
Education level, *n* (%)					< 0.001
Lower than high school	2107 (27.08)	419 (25.50)	1032 (27.41)	656 (27.66)	
High school	1758 (22.60)	363 (22.09)	870 (23.11)	525 (22.13)	
Some college or AA degree	2311 (29.70)	416 (25.32)	1296 (34.42)	599 (25.25)	
College graduate or above	1604 (20.62)	445 (27.08)	567 (15.06)	592 (24.96)	
Family PIR^1^, *n* (%)					< 0.001
<= 1.3	2560 (32.90)	568 (34.57)	717 (30.23)	1275 (33.86)	
1.3 ~ 3.5	3201 (41.14)	645 (39.26)	924 (38.95)	1632 (43.35)	
> 3.5	2560 (32.90)	430 (26.17)	731 (30.82)	858 (22.79)	
Marital status, *n* (%)					< 0.001
Married	4035 (51.86)	735 (44.74)	1411 (59.49)	1889 (50.17)	
Other	3745 (48.14)	908 (55.26)	961 (40.51)	1876 (49.83)	
Country of birth, *n* (%)					< 0.001
US born	5678 (72.98)	1104 (67.19)	1579 (66.57)	2995 (79.55)	
Non-US born	2102 (27.02)	539 (32.81)	793 (33.43)	770 (20.45)	
Alcohol use status (yes), *n* (%)	1214 (15.60)	242 (14.73)	373 (15.73)	599 (15.91)	0.536
Hypertension (yes), *n* (%)	4606 (59.2)	701 (42.67)	1326 (55.90)	2579 (68.50)	0.144
Smoking status, *n* (%)					< 0.001
Current smoker	1457 (18.73)	382 (23.25)	354 (14.92)	721 (19.15)	
Former smoker	2347 (30.17)	365 (22.22)	840 (35.41)	1142 (30.33)	
Never smoker	3976 (51.11)	896 (54.53)	1178 (49.66)	1902 (50.52)	
Physical activity, *n* (%)					0.321
High	1242 (15.96)	244 (14.85)	394 (16.61)	604 (16.04)	
Low	6538 (84.04)	1399 (85.15)	1978 (83.39)	3161 (83.96)	
Diabetes (yes), *n* (%)	2558 (32.88)	260 (15.82)	672 (28.33)	1626 (43.19)	< 0.001
Energy intake (kcal/day); median (IQR)	1908.5 (750.82)	1871.03 (732.31)	1945.75 (739.13) ^a***^	1901.69 (759)	0.008
Time of blood draw, *n* (%)					< 0.001
Morning	3825 (49.16)	877 (53.38)	1136 (47.89)	1812 (48.13)	
Afternoon	2968 (38.15)	581 (35.36)	963 (40.60)	1424 (37.82)	
Evening	987 (12.69)	185 (11.26)	273 (11.51)	529 (14.05)	

7780 participants were divided into three subgroups based on body mass index: ‘Normal weight’, ‘Overweight’, and ‘Obese’. Mann-Whitney U test and Kruskal-Wallis’ rank sum test were used to compare the differences in continuous variables between subgroups. Chi-square test was used to find differences in categorical variables between subgroups.

a: Compared to the ‘Normal weight’ subgroup.

b: Compared to the ‘Overweight’ subgroup.

*P< 0.05, **P< 0.01 and ***P< 0.001.

^1^ Family PIR represents the ratio of family income to poverty.

**Table 2** characterizes the levels and distribution of serum sex steroid hormones (TT, estradiol and SHBG), urinary creatinine and urinary phthalate metabolites by median and interquartile spacing (IQR). We used the Kruskal-Wallis’ rank sum test to compare between-group differences in continuous variables across the three subgroups, and the Mann-Whitney U test for two-by-two comparisons between subgroups. We found significant differences in the levels and distributions of these laboratory variables across the three subgroups, with the results of the Mann-Whitney U test showing more pronounced differences between the subgroups ‘Normal weight’ and ‘Obese’. Median urinary levels of phthalate metabolites were significantly higher in the ‘Obese’ subgroup than in the other two subgroups, as were median levels of urinary creatinine (115.0 mg/dL). Serum estradiol levels were higher in the ‘Obese’ subgroup (21.3 pg/mL), whereas serum SHBG levels were instead lower (45.5 nmol/L). Remarkably, the median level of serum TT was much higher in the ‘Overweight’ subgroup (268.0 ng/dL) than in the ‘Normal weight’ and ‘Obese’ subgroups. Weighted statistical descriptions of serum sex steroid hormones, urinary creatinine, and urinary phthalate metabolites are shown in **Supplementary Table 2**.

**Table 2:** Descriptive statistics for phthalate metabolites and sex steroid hormones in different subgroups.

	Overall	Normal weight	Overweight	Obese	*P* values
*n* (%)	7780 (100)	1643 (21.1)	2372 (30.5)	3765 (48.4)	
Urinary creatinine (mg/dL)	107.0 (102.0)	91.0 (100.0)	103.0 (98.0) ^a***^	115.0 (105.0) ^a***,b***^	< 0.001
Phthalates (ng/mL) ^#^					
MBP	11.0 (15.4)	10.9 (16.7)	9.7 (14.6)	11.9 (15.4) ^a***,b***^	< 0.001
MBzP	3.7 (7.7)	3.7 (7.4)	3.2 (5.9) ^a*^	4.0 (8.7) ^a***,b***^	< 0.001
MCNP	1.9 (2.8)	1.8 (2.5)	1.8 (2.4)	2.1 (3.0) ^a***,b***^	< 0.001
MCOP	10.15 (24.3)	8.0 (20.3)	9.9 (20.3) ^a*^	12.5 (30.2) ^a***,b***^	< 0.001
MCPP	1.3 (2.5)	1.3 (2.3)	1.1 (2.3)	1.5 (2.8) ^a***,b***^	< 0.001
MECPP	10.4 (13.9)	10.1 (14.9)	9.4 (12.6)	11.4 (14.4) ^a***,b***^	< 0.001
MEHHP	6.9 (10.0)	6.4 (10.8)	5.8 (8.9)	7.7 (10.1) ^a***,b***^	< 0.001
MEHP	1.1 (1.7)	1.1 (1.8)	1.0 (1.8) ^a*^	1.1 (1.6) ^a***^	0.033
MEOHP	4.4 (6.5)	4.3 (7.2)	3.8 (5.4)	4.7 (6.5) ^a***,b***^	< 0.001
MEP	38.05 (104.1)	30.6 (83.6)	33.25 (93.2)	44.9 (127.6) ^a***,b***^	< 0.001
MiBP	8.5 (12.4)	7.6 (11.7)	8.1 (11.5) ^a*^	9.2 (13.9) ^a***,b***^	< 0.001
Serum sex hormones					
Total testosterone (ng/dL)	85.0 (333.1)	37.44 (402.4)	268.0 (375.9) ^a***^	43.8 (276.7) ^a***,b***^	< 0.001
Estradiol (pg/mL)	20.55 (23.8)	18.6 (30.0)	20.9 (20.3) ^a**^	21.3 (24.1) ^a***,b***^	< 0.001
SHBG (nmol/L)	51.53 (35.3)	68.96 (55.2)	53.63 (32.9) ^a***^	45.54 (28.8) ^a***,b***^	< 0.001

Mann-Whitney U test and Kruskal-Wallis’ rank sum test were used to compare the differences between subgroups. a: Compared to the ‘Normal weight’ subgroup.

b: Compared to the ‘Overweight’ subgroup.

*P< 0.05, **P< 0.01 and ***P< 0.001.

^#^See **Supplementary Table 4** for full names of abbreviated phthalate metabolites.

### 3.2 Regression results

**Figure 2:**
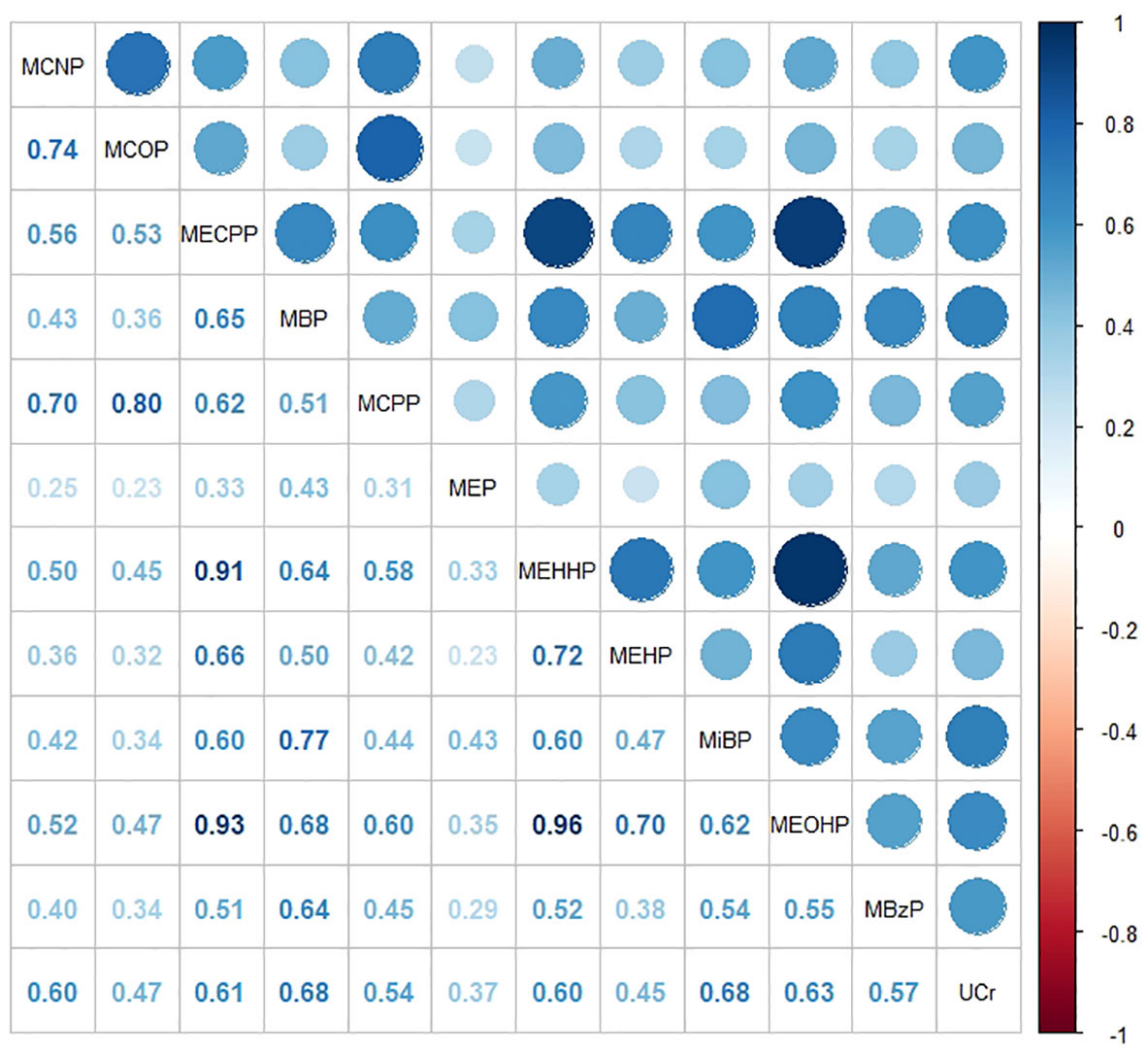
Heat map showing Spearman’s correlation matrix for concentrations of eleven urinary phthalate metabolites and urinary creatinine levels. The FDR-corrected *P* values indicate that Spearman’s correlation matrix is statistically significant (*P< 0.001)*. The color corresponds to the strength of correlations (blue: positive correlation; white: no correlation; red: negative correlation).

To examine the distinct associations among PAEs metabolites, sex steroid hormones, and participants’ obesity status, we constructed a multinomial logistic regression model with obesity status as a three-categorical dependent variable. However, we observed multicollinearity among these phthalate metabolites. Additionally, since these metabolites were sampled in urine samples, there was some correlation with urinary creatinine levels. We generated correlation heatmaps to illustrate the relationships between PAEs metabolites and urinary creatinine levels, as depicted in **Figure 2**. Positive correlations between PAEs metabolites and urinary creatinine were prevalent, and the FDR-corrected *P*-values remained significant (*P< 0.001*).

To address the issue of multicollinearity among phthalate metabolites, we employed principal component analysis (PCA) to reduce the dimensionality of the original variables. As shown in **Supplementary Figure 1**, PCA extracted 11 principal components that accounted for all the variances in the original variables. Notably, the proportions of variances explained by the components beyond the sixth were consistently lower than 0.04. Consequently, we opted for the first six principal components instead of the original variables, collectively explaining 90.5% of the total variance in the original variables. The contribution of each phthalate metabolite to each PC is presented in **Supplementary Table 3**. These principal components derived from PCA were incorporated into multinomial logistic regression to calculate odds ratios (ORs) and corresponding 95% confidence intervals (CI) for the other two categorical endpoints, with ‘Normal weight’ as the reference (**Table 3**). The regression results revealed a statistically significant association between PC1, PC2, and obesity status, with PC2 posing a risk factor for obesity (OR = 1.082, *P*< 0.001) and PC1 acting as a protective factor (OR = 0.890, *P* = 0.003). When using ‘Normal weight’ as the reference, PC5 emerged as a risk factor for the population’s tendency to be overweight (OR = 1.152, *P* = 0.02), but the association with obesity was not statistically significant (*P* = 0.136). Since principal components can be interpreted as linear combinations of primitive continuous variables, those associated with obesity status can be analyzed based on their composition. As outlined in **Table S3**, the risk factor PC2 for obesity status was primarily explained by MCNP, MCOP, and MCPP, while the protective factor PC1 was predominantly explained by MECPP, MBP, MEHHP, MEHP, and MEOHP. Concerning sex steroid hormones, TT and SHBG emerged as protective factors for obesity status (OR = 0.997; OR = 0.977), with only estradiol identified as a risk factor (OR = 1.008). Among other covariates, hypertension and diabetes were significant risk factors for obesity (OR = 2.938; OR = 3.31).

**Table 3:** Results of multinomial logistic regression.

	Overweight	Obese
**Age**	**1.015 (1.012, 1.019)**	1.001 (0.997, 1.004)
Gender		
male	1.000 (reference)	1.000 (reference)
female	**0.749 (0.710, 0.790)**	**0.869 (0.822, 0.918)**
Race		
Mexican American	1.000 (reference)	1.000 (reference)
Non-Hispanic White People	**0.492 (0.453, 0.535)**	**0.353 (0.323, 0.385)**
Non-Hispanic Black People	**0.716 (0.673, 0.762)**	**0.480 (0.451, 0.510)**
Other Hispanic	1.007 (0.932, 1.087)	**0.728 (0.676, 0.783)**
Other Race	**0.312 (0.288, 0.339)**	**0.129 (0.119, 0.140)**
Education		
Lower than high school	1.000 (reference)	1.000 (reference)
High school	0.981 (0.925, 1.040)	0.964 (0.906, 1.025)
Some college or AA degree	1.039 (0.986, 1.095)	**1.290 (1.217, 1.367)**
College graduate or above	**0.831 (0.779, 0.887)**	**0.686 (0.644, 0.731)**
Family PIR^1^		
<= 1.3	1.000 (reference)	1.000 (reference)
1.3 ~ 3.5	**0.928 (0.863, 0.998)**	**1.097 (1.018, 1.183)**
> 3.5	**1.227 (1.141, 1.320)**	1.069 (0.995, 1.149)
Marital status		
Other	1.000 (reference)	1.000 (reference)
Married	**0.671 (0.628, 0.716)**	**0.748 (0.699, 0.799)**
Country of birth		
US born	1.000 (reference)	1.000 (reference)
Non-US born	**0.881 (0.805, 0.965)**	**0.411 (0.377, 0.450)**
Alcohol using		
No	1.000 (reference)	1.000 (reference)
Yes	**0.891 (0.824, 0.964)**	**0.883 (0.816, 0.956)**
Hypertension		
No	1.000 (reference)	1.000 (reference)
Yes	**1.576 (1.476, 1.682)**	**2.938 (2.753, 3.134)**
Diabetes		
No	1.000 (reference)	1.000 (reference)
Yes	**1.506 (1.409, 1.608)**	**3.310 (3.092, 3.543)**
Physical activity		
Low	1.000 (reference)	1.000 (reference)
High	1.031 (0.954, 1.114)	**1.130 (1.044, 1.222)**
Smoking status		
Current smoker	1.000 (reference)	1.000 (reference)
Former smoker	**1.558 (1.454, 1.669)**	**1.425 (1.334, 1.523)**
Never smoker	**0.854 (0.792, 0.922)**	1.005 (0.924, 1.094)
**Energy intake^2^**	**1.000 (1.000, 1.000)**	**1.000 (1.000, 1.000)**
**Urinary creatinine**	**1.005 (1.004, 1.006)**	**1.006 (1.005, 1.007)**
Principal components of PAEs^3^		
PC 1	**0.852 (0.819, 0.887)**	**0.890 (0.857, 0.926)**
PC 2	**1.069 (1.013, 1.128)**	**1.082 (1.027, 1.141)**
PC 3	0.964 (0.903, 1.029)	1.054 (0.989, 1.124)
PC 4	1.010 (0.937, 1.090)	0.971 (0.903, 1.045)
PC 5	**1.152 (1.053, 1.260)**	1.067 (0.980, 1.163)
PC 6	1.033 (0.934, 1.143)	1.023 (0.926, 1.129)
Time of blood draw		
Morning	1.000 (reference)	1.000 (reference)
Afternoon	**1.323 (1.241, 1.410)**	1.037 (0.973, 1.105)
Evening	**1.287 (1.183, 1.402)**	**1.458 (1.334, 1.594)**
Sex steroid hormone		
Total testosterone	**0.999 (0.999, 1.000)**	**0.997 (0.997, 0.998)**
Estradiol	**1.005 (1.002, 1.007)**	**1.008 (1.006, 1.011)**
SHBG	**0.985 (0.983, 0.987)**	**0.977 (0.975, 0.979)**

The results of the multinomial logistic regression are expressed as ORs and 95% CIs, with ‘Normal weight’ as the reference. Bolded ORs indicate statistical significance (P<0.05). The categorical variables are referenced to the selected categories and the corresponding ORs are obtained.

^1^Family PIR represents the ratio of family income to poverty.

^2^Average daily energy intake (kcal/day).

^3^Principal components (PC) consisting of the original PAEs variables.

The WQS regression method was employed to examine the impact of mixed PAEs exposure on sex steroid hormones and BMI (as a continuous variable). This weighted approach aimed to amalgamate PAEs metabolites into a ‘phthalate index’ to address multicollinearity among the original variables. The objective was to obtain interpretable regression coefficients that quantify the combined effect of phthalate metabolites on sex hormones. The WQS regression results indicated that the phthalate index was significant for all three sex steroid hormones and BMI (**Table 4**). Notably, the phthalate index exhibited a negative correlation with TT (*β* = -20.85, *P< 0.001*) and positive correlations with estradiol (*β* = 3.00, *P = 0.001*) and SHBG (*β* = 5.22, *P< 0.001*). Regarding the negative correlation of the phthalate index with total testosterone, MCOP contributed the most with 34.6%, while in the positive correlation with estradiol, MiBP contributed the most with 37.0%. In the positive correlation of the phthalate index with SHBG, MEOHP accounted for the most with 24.3% (**Table 5**). Additionally, the PAEs index demonstrated a positive correlation with BMI, with MEP contributing to 41.0% of the mean weight.

**Table 4:** Results of weighted quantile sum regression.

	Phthalates index*	*P* values
Total testosterone	-20.85 (-31.62, -10.08)	< 0.001
Estradiol	3.00 (1.21, 4.79)	0.001
SHBG	5.22 (2.17, 8.27)	< 0.001
BMI	0.52 (0.15, 0.90)	0.007

*Regression coefficients and 95% CI for mixed exposures in the weighted quantile sum regression.

**Table 5:** Composition of phthalate metabolites in mixed exposures.

Phthalates index	Total testosterone	Estradiol	SHBG	BMI
MCNP	0.072	0.048	0.152	0.070
MCOP	0.346*	0.076	0.011	0.120
MECPP	0.043	0.032	0.008	0.110
MBP	0.108	0.029	0.023	0.000
MCPP	0.001	0.136	0.022	0.039
MEP	0.109	0.130	0.082	0.410*
MEHHP	0.181	0.008	0.165	0.048
MEHP	0.058	0.121	0.067	0.000
MiBP	0.013	0.370*	0.193	0.000
MEOHP	0.069	0.031	0.243*	0.140
MBzP	0.000	0.021	0.033	0.065

*Original variables with the largest contribution to the Phthalates index.

Given the variation in sex hormone levels across genders and ages, we conducted subgroup analyses to explore potential differences (**Supplementary Table 5**). For age stratification, participants were divided into ‘middle-aged’ and ‘older’ subgroups using the median age (60 years) as the cutoff. The stable negative correlation between PC1 and obesity was observed across all four subgroups, with no statistically significant differences in ‘Female-mid’ and ‘Male-mid’. WQS regression was applied to investigate the effects of mixed PAEs exposure on sex hormones in all subgroups (**Supplementary Table 6**). The results revealed a positive association between BMI and mixed PAEs exposure in all subgroups, along with a negative association between TT and mixed PAEs exposure. Concerning estradiol, a positive correlation with mixed PAEs exposure was observed in all subgroups except for ‘Male-mid’. Similarly, a positive correlation with mixed PAEs exposure was noted for SHBG in all subgroups, though the differences in ‘Female-mid’ and ‘Male-mid’ were not statistically significant.

### 3.3 Machine learning models

After obtaining regression results, our next objective was to discern the predominant influences of sex hormones and phthalate metabolites on the population’s obesity status. To achieve this, we employed various algorithms for prediction models. Independent variables underwent preprocessing using the one-hot coding technique, as some machine learning algorithms do not accommodate categorical independent variables. The total samples were randomly divided into an 80% training set and a 20% validation set. Utilizing the grid search technique, the training set entered a suitable hyperparameter space to identify the optimal hyperparameter combination. To mitigate errors from random sampling, a 5-fold cross-validation was employed during the search process. This methodology pinpointed the hyperparameter combination minimizing the average error, used to construct the prediction model. Subsequently, the validation set evaluated the predictive performance of the model, and the ‘Accuracy’ represented the ratio of correctly predicted samples to the total number in the validation set. F1-score is obtained from the confusion matrix of the prediction results, which is a comprehensive evaluation combining ‘Precision’ and ‘Recall’, and is calculated by the formula of $F1=\frac{2\times Precision\times Recall}{Precision+Recall}$ . Simply put, when the F1-score is higher, the accuracy and recall are higher and the model has better predictive power.

When comparing the predictive performance of all models (**Table 6**), those with high accuracy also exhibit higher F1-scores. To ensure an unbiased model selection, we introduced several simple yet classical algorithms, including KNNs, Naive Bayes, and the acquired multinomial logistic regression model. Regression models and Naive Bayes, being less reliant on hyperparameter tuning, are user-friendly and easily interpretable. However, their prediction accuracy, as revealed by the outcomes, falls below 60%, indicating suboptimal efficacy on our dataset. The KNN algorithm achieves a prediction accuracy of 83.4% with an F1-score of 0.813, employing a hyperparameter ‘k’ set to 1. SVM, classified into ‘radial’ and ‘polynomial’ based on different kernel functions, both demonstrate an accuracy of approximately 85%, with F1-scores exceeding 0.83. Despite the decision tree model having an accuracy of 80.4%, the decision tree algorithm remains a foundational concept for numerous complex algorithms.

**Table 6:** Classification performance of all models.

	hyper-parameters	Accuracy	F1-score
multinomial logistic regression	–	0.586	0.530
Naive Bayes	–	0.530	0.517
KNN^1^	k = 1	0.834	0.813
Support vector machine			
radial	kernel = ‘radial’; degree = 5; cost = 25; gamma = 0.01;	0.846	0.835
polynomial	kernel = ‘polynomial’; degree = 5; cost = 3.667; gamma = 1.117;	0.851	0.835
Decision Tree	minsplit = 1; minbucket = 2; cp = 0.001; maxdepth = 23;	0.804	0.784
Random Forest	ntree = 300; mtry = 15; nodesize = 12; maxnodes = 350;	0.882	0.870
GBDT^2^	distribution = ‘multinomial’; n.trees = 400; n.minobsinnode = 40; shrinkage = 0.9;	0.771	0.752
XGBoost^3^	eta = 0.1; gamma = 0.556; max_depth = 10; min_child_weight = 1.5; colsample_bytree = 0.5; nrounds = 100; eval_metric = ‘mlogloss’;	0.891	0.879

The classification models were built using machine learning algorithms with adjusted parameters, and accuracy and F1-score were used as model evaluation metrics.

^1^KNN represents K Nearest Neighbor.

^2^GBDT represents Gradient Boosting Decision Tree.

^3^XGBoost represents eXtreme Gradient Boosting.

The RF algorithm is an extension of the decision tree classification algorithm. In our RF model, we utilize 300 decision tree models (ntree = 300), a maximum of 15 features used on the nodes of each decision tree model (mtry = 15), a minimum of 12 samples on the leaf nodes (nodesize = 12), and a total maximum number of leaf nodes set to 350 (maxnodes = 350). With this set of hyperparameters, the RF model achieves an accuracy of 88.4% and an F1-score of 0.87. Despite the randomization method employed by RF, which reduces the risk of overfitting, we exercise control over the values of mtry, nodesize, and maxnodes to further mitigate the potential for overfitting during model training.

**Figure 3:**
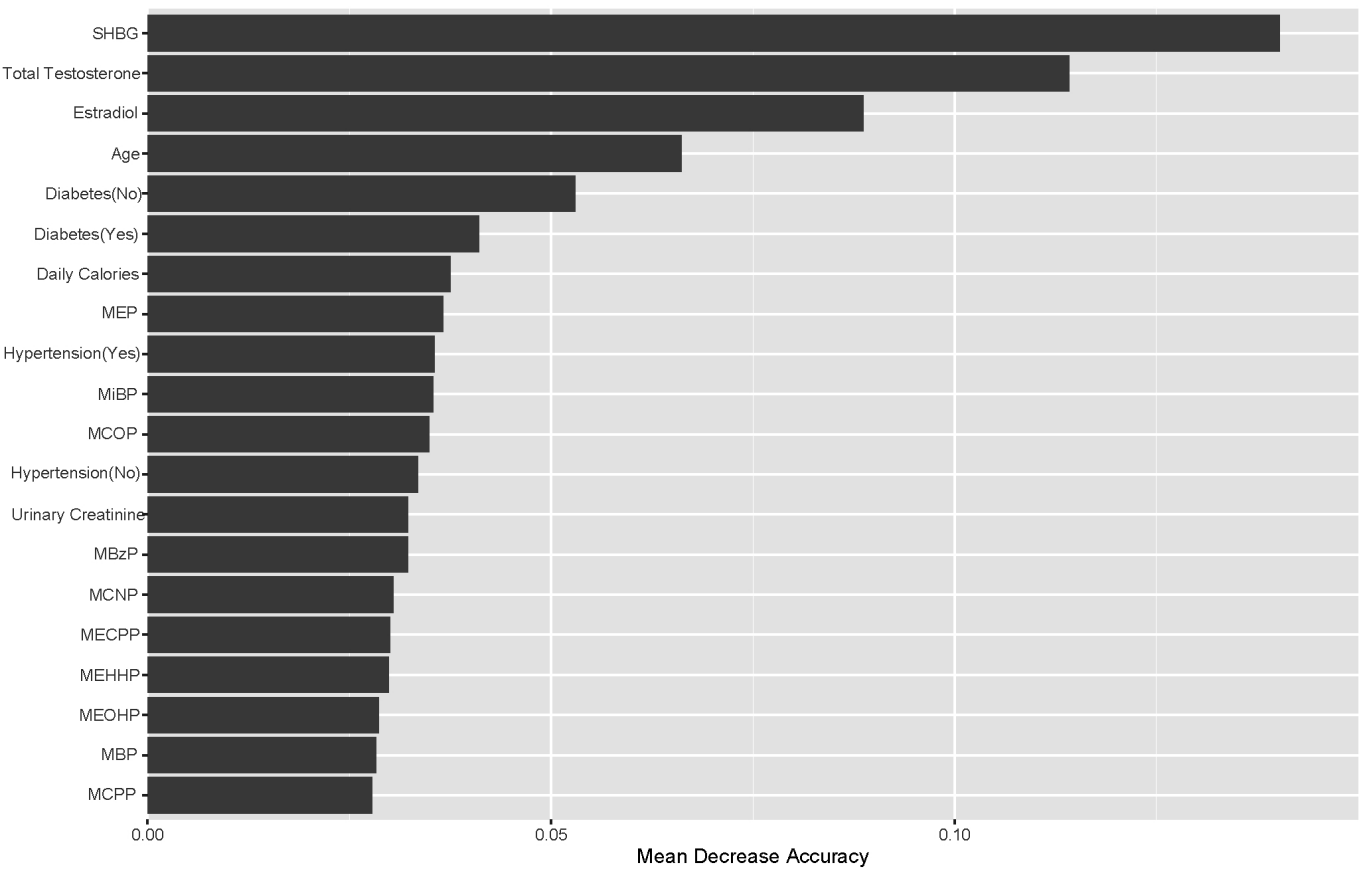
Top-20 importance ranking features based on mean decrease accuracy from the RF model. Among PAEs metabolites, MEP, MiBP and MCOP exhibited higher mean decreasing accuracy values.

The importance of the characteristics in the RF model for the three categorical endpoints was comprehensively evaluated using ‘mean decrease accuracy’ (41). The top 20 independent variables contributed to 83% of the mean decrease accuracy values of all variables (**Figure 3**). The sex hormone variable emerged as the most significant contributor to the predictive accuracy of the model, followed by age, diabetes mellitus, average daily caloric intake, hypertension, and phthalate metabolite levels. These findings underscore the importance of age, diabetes, and hypertension as crucial factors in predicting obesity status. Furthermore, the substantial variations in sex hormone levels across different subsets highlight the significant contributions of these three sex hormone variables to the predictions of the RF model. Among PAEs metabolites, MEP, MiBP and MCOP exhibited higher mean decreasing accuracy values (**Figure 3**).

Both GBDT and RF are extensions of the decision tree algorithm, but RF is a variant of the decision tree algorithm optimized with the bootstrap aggregating (or bagging for short) technique, while GBDT is the decision tree algorithm optimized with the gradient boosting technique. Since our target variable involves a triple classification of physical states, the hyperparameter ‘distribution’ for GBDT is set to ‘multinomial’. The final GBDT model consists of a total of 400 decision trees (n.trees = 400), with a minimum of 40 observations in the terminal nodes (n.minobsinnode = 40), and a learning rate of 0.9 for each decision tree (shrinkage = 0.9). However, the GBDT model’s prediction accuracy is only 77.1%, and the F1-score is only 0.752, indicating suboptimal performance on our dataset. In response to this, we explored the XGBoost algorithm, which is also grounded in the gradient boosting technique.

In our XGBoost model, the learning rate is set to 0.1 (eta = 0.1), the minimum loss reduction at leaf nodes is 0.556 (gamma = 0.556), the maximum depth of the trees is 10 (max_depth = 10), the minimum impurity level before node division is 1.5 (min_child_weight = 1.5), the proportion of independent variables used in a single decision tree is 0.5 (colsample_bytree = 0.5), the total number of decision trees is 100 (nrounds = 100), and the loss function employed is the logarithmic loss function (eval_metric = ‘mlogloss’). We also constrained the search range of hyperparameters during the hyperparameter search to prevent overfitting. This configuration of hyperparameters for our XGBoost model yields a prediction accuracy of 89.1% and an F1-score of 0.879.

**Figure 4:**
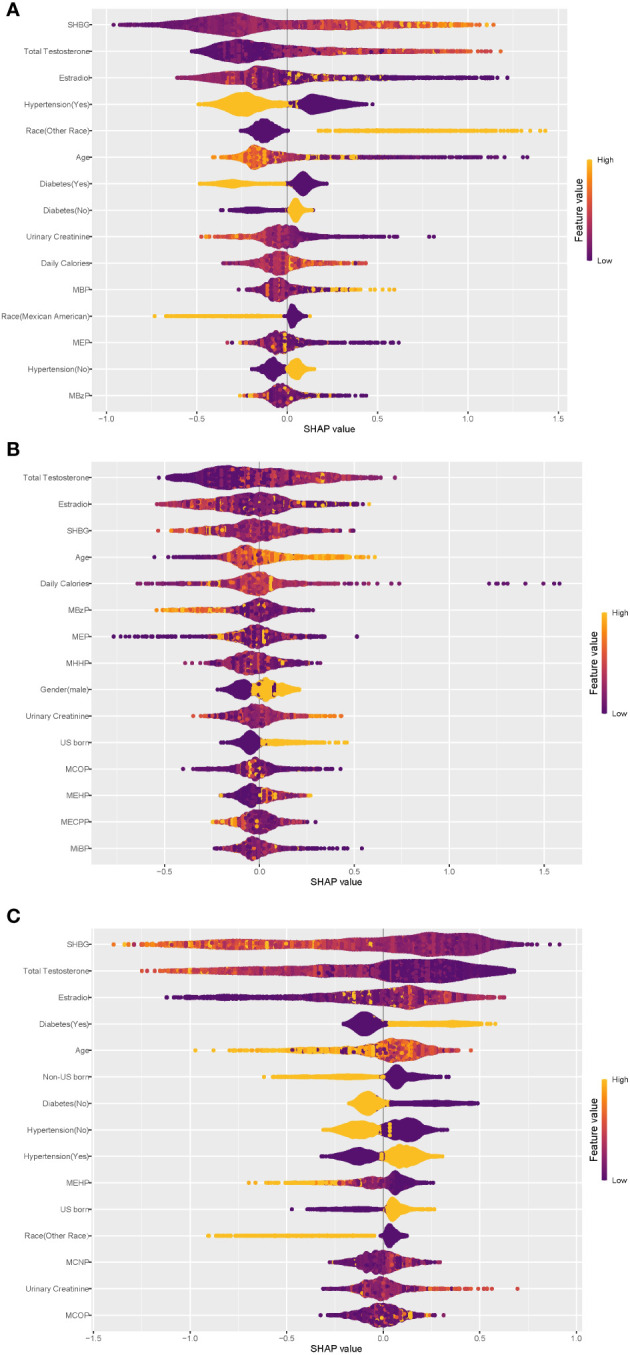
Global explainability of physical state. Global explainability of the XGBoost model for the top 15 most important variables (ranked in order of importance based on the mean of the absolute SHAP values). Each dot color codes the SHAP value of each variable for each individual; yellow and purple indicate high and low values of the variable, respectively. A positive or negative SHAP value on the x-axis imply that the variable contributes to a positive or negative estimate of physical state for a given individual. **(A)** Global explainability of normal weight state. **(B)** Global explainability of overweight state. **(C)** Global explainability of obesity state.

To assess the contribution of each variable in predicting individual physical states, we applied the Shapley additive explanatory (SHAP) tree framework to the XGBoost model with a customized loss model (42). The SHAP value combines the effect of a given variable on its own and the effect of the interaction of that variable with other parameters. For a given individual (local interpretation), the sum of the SHAP values for all variables of the model represents the deviation of the individual from the predicted propensity of obesity status for the entire dataset. The greater the overall SHAP value, the more significant the contribution of the variable to predicting obesity status. The global SHAP values for the top 15 variables, as depicted in **Figure 4**, account for 73.2%, 67.9%, and 71.2% of the average total SHAP contribution, respectively. The three subplots depict the global SHAP values for Normal weight (**Figure 4A**), Overweight (**Figure 4B**), and Obese (**Figure 4C**) respectively.

As depicted in **Figure 4**, within the XGBoost model, sex steroid hormones exhibited the most substantial contributions to predicting all obesity conditions. Additionally, age, hypertension, diabetes, urinary creatinine, and certain phthalate metabolites showed high global SHAP values. Notably, individuals with hypertension or diabetes displayed a clear inclination toward obesity, underscoring the significant role of hypertension and diabetes as risk factors for obesity, consistent with earlier regression findings. Among the top 15 variables contributing most to the prediction of obesity status (**Figure 4C**), only three phthalate metabolites—MEHP, MCNP, and MCOP—were present, with MEHP being consistently negatively associated with obesity. Diabetes exhibited a negative association with ‘Normal weight’ status (**Figure 4A**). Participants from ‘other races’ tended to predict normal weight, while Mexican Americans displayed the opposite trend. The regression results from the principal component analyses described earlier indicated statistically significant associations between the three sex steroid hormones and obesity status, with a low strength of association (odds ratios approximating 1.0), consistent with the global SHAP values for the sex hormone variables in **Figure 4**.

## 4 Discussion

The analysis results unveiled noteworthy connections between sex steroid hormones and obesity in the population, with specific principal components of phthalate metabolite composition also displaying substantial associations with obesity status in US adults. The outcomes from WQS regression models pointed out that mixed exposure to phthalate metabolites was linked to total testosterone TT, estradiol, and SHBG. Among various predictive models, RF and XGBoost exhibited superior predictive performance for obesity, with sex steroid hormones contributing the most to the model predictions, followed by demographic variables such as diabetes and hypertension, and phthalate metabolites.

PAEs, as a typical environmental EDCs, have multifaceted and multi-systemic effects on human health (43). This study reveals a positive correlation between PAEs exposure and BMI, with MEP contributing 41.0% to the average weight of PAEs index. The regression findings indicated a positive connection between the PC2 of phthalate metabolites and the likelihood of obesity. MCNP, MCOP, and MCPP constituted the primary contributing factors to PC2, thus categorizing them as obesity risk factors. Additionally, the fifth principal component (PC5), predominantly composed of MiBP and MBP, displayed a positive correlation with the occurrence of overweight. These findings underscore the complex and interconnected impact of PAEs on human health, particularly in relation to weight-related outcomes. These results align closely with previous research findings. Stahlhut et al. found the PAEs exposures and their associations with obesity in adult US males (participant in the NHANES 1999-2002) (43). MBP, MCOP, MCNP, MCPP and MECPP were also found to be associated with obesity or BMI in adults participating in the U.S.-based NHANES (44, 45). Notably, MCOP exhibited associations not only with BMI but also with waist circumference. In a more recent cohort study involving 942 elderly individuals in China(Li et al.) (46), urinary levels of MEP, MEOHP, MBP, and MMP were positively associated with general obesity in males. Furthermore, an intriguing discovery indicated that MCNP, MCOP, and MEHP play a role in the onset of obesity (47). A comprehensive meta-analysis concluded that MMP, MEP, and MiBP showed positive associations with abdominal obesity, while MEHHP, MECPP, and MCOP exhibited positive correlations with general obesity in adults (48) Their study formed part of a broader understanding of phthalate exposure and its effects on obesity-related outcomes. In a longitudinal cohort study conducted by the Women’s Health Initiative (WHI), certain phthalate biomarkers, including MCNP, were found to be positively associated with an increase in visceral adipose tissue (VAT) in postmenopausal women. However, no significant correlation was established between other phthalate biomarkers (MCOP, MCPP, etc.) and either VAT or subcutaneous adipose tissue (SAT) (49). This cumulative evidence supports the intricate relationship between phthalate exposures and obesity across diverse populations and study designs.

Sexual steroid hormones affect the metabolism, distribution, and increase of adipose tissue by binding to receptors in adipose tissue, and a decrease in estrogen and/or androgens typically leads to central obesity (50). The reproductive toxicity of PAEs, as confirmed by numerous existing studies, encompasses adverse effects on the HPG axis, including abnormal release of gonadotropin-releasing hormone and gonadotropins, along with dysfunction of sex hormone receptors and steroid hormone synthesis (23). These factors collectively contribute to a heightened prevalence of metabolic disorders (38, 51). Additionally, some researchers contend that obesity, on the contrary, heightens the risk of sex hormone imbalances (52). Lapauw et al. found that low serum SHBG and total testosterone levels were very common in obese men (53). Our results indicated that estradiol was positively associated with obesity, while total testosterone and SHBG levels were negatively associated with obesity. The relationship between these elevated rates and the concomitant presence of chronic sex hormone imbalances and PAEs exposure warrants further investigation and scrutiny.

Building upon this, it’s noteworthy that combined exposure to phthalates and their metabolites contributes to an elevation in estradiol and SHBG levels, coupled with a reduction in TT levels. The disruptive impact of phthalates on hormonal balance has been established in both animal and human studies, suggesting potential implications for endocrine systems. In particular, DEHP has exhibited anti-androgenic effects and estrogen-mimicking activities both *in vivo* and *in vitro*, and it has been associated with decreased TT levels in male animals and humans (54–57). Cathey et al. found that TT was positively associated with MHBP and inversely associated with MEP in women during pregnancy (57). Similarly, urinary MEHP was found to be inversely associated with circulating steroid hormone levels in adult men (58). Drawing on data from NHANES 2015-2016, a study involving 1768 adults measured 16 urinary phthalate metabolites and three serum sex hormones. Among males, TT levels displayed a negative association with MnBP, MEHHP, MECPP, MEP, and MiBP. Conversely, among females, the natural logarithm-transformed estradiol exhibited an increase of 0.18 pg/mL and 0.15 pg/mL with each 1 natural logarithm-concentration rise in MEHP and MNP, respectively (30). In a cross-sectional study involving 614 women aged 45-54 years, an association was identified between phthalate exposure and an increase in estradiol levels (59). However, when comparing the relationship between SHBG and phthalate metabolites, disparities emerged between previous studies and our current research. Some studies reported that elevated levels of exposure to MECCP, MEOHP, MEHHP, and MBzP were linked to decreased SHBG levels, but not to increased TT levels (58). We attribute these variations to differences in the study population and our comprehensive approach to phthalate exposure analysis.

In the negative correlation of phthalate index with TT, MCOP contributed the most (34.6%), while in the positive correlation of phthalate index with estradiol, MiBP contributed the most with 37.0%. MEOHP accounted for the most in the positive correlation of phthalate index and SHBG with 24.3%. The correlation between TT and MCOP aligns with findings from previous research. A study focusing on midlife women revealed a negative correlation between MCOP and TT levels. (D%: -2.08%; 95% CI, -3.66 to -0.47) (60). Further, a study involving 1179 children aged 6-19 years demonstrated that MiBP, MCOP and MBzP were generally negatively associated with estrodial and TT, while positively associated with SHBG (61). However, the correlation between estrogen and MiBP shows some differences compared to previous studies. MCOP and MBzP exhibited a positive association with estrogen, while MEP, MiBP, and MEOHP demonstrated an inverse correlation with estrogen (62). In a study focused on 297 women of childbearing age, MiBP was linked to a 0.01 (95% CI: -0.01, 0.00) decrease in natural logarithm-unit levels of estradiol. Additionally, a study involving 297 girls aged 12 to 19 in the NHANES (2013-2016) found that MBzP was positively associated with SHBG, while MCNP and MECPP showed an inverse association with SHBG (62).

In this study, variations in the influence of PAEs metabolites on estrogen and SHBG were observed compared to the existing literature. To further investigate the reasons behind the aforementioned differences, we stratified the study population based on age and gender. In group “Male-mid”, we observed a negative correlation between PAEs exposure and estrogen levels, while in the other subgroups, it showed a positive correlation. This finding aligns with results reported in certain earlier studies. In male adolescents, there was a negative correlation between PAEs and estrogen (β= -0.137, 95% CI: -0.263, -0.011), as well as TT (β= -0.189, 95% CI: -0.375, -0.002) (63). Data from U.S. population found that exposure to PAEs, both individuals and as a mixture, was inversely associated with estradiol levels and the ratio of TT to estradiol in children (61). In a cross-sectional investigation, it was observed that urinary DEHP metabolites and MEHP exhibited a notable positive correlation with serum estradiol levels (64). This aligns with a previous study, which indicated a positive correlation between urinary DEHP metabolites (MEHP, MEOHP, MEHHP) and estradiol levels in polyvinyl chloride production workers (65). On the contrary, DEHP was linked to reduced serum estradiol levels in postmenopausal women (36), a finding corroborated by prior studies that reported non-significant negative results (66, 67). Gender-specific analyses indicated that phthalate exposure has a distinct impact on various sex steroid hormones. Exposure to phthalates (PAEs) manifests most prominently in sex hormones among middle-aged individuals, while it exhibits a more pronounced effect on BMI increase in elderly females.

To explore the association between phthalate metabolites and obesity in the population from the perspective of sex hormones, we used multiple machine learning algorithms to model the data of these algorithms from the studied cohort. Of these algorithms, RF and XGBoost exhibited the best classification performance. During the interpretation of the models, we observed that sex steroid hormones tended to perform better. Hypertensive and diabetic individuals had a higher risk of obesity, consistent with the regression results. Phthalate metabolites also contributed significantly to the model classification performance. A relatively clear negative correlation was found, during the interpretation of the XGBoost model, between MEHP and population obesity. However, a study by Desvergne et al. found that MEHP, a selective PPARγ modulator, is capable of disrupting lipid and carbohydrate metabolism, thereby increasing the risk of obesity (68). For the negative association with obesity exhibited by MEHP in this study, we speculate that it is related to the multiple correlations of this class of phthalate metabolites. Considering that the hydrolysis products of DEHP are sufficiently complex, perhaps MEHP could serve as an intermediate cue for the DEHP hydrolysis process, which requires more research to elucidate the mechanisms involved.

In this study, we used principal component analysis to deal with the issue of collinearity PAEs metabolites and combined with sex hormone status to explore the association between PAEs and obesity in the population. We tried multiple classes of predictive models, and the results showed that XGBOOST and RF performed better; such integrated models have good interpretability and can fully exploit potentially meaningful associations among numerous features. It is hoped that integrated machine learning algorithms will be considered and attempted in a wide range of bioinformatic research. This study has certain limitations. Firstly, adjustment methods for urine dilution could bias conclusion. As urinary creatinine levels may be affected by factors including age, sex, and kidney disease, statistical estimation with traditional creatinine adjustment may be influenced under certain circumstances (69). The use of urinary creatinine to adjust urine dilution may bias the chemical exposure estimates and therefore the association with the health outcome as well. And we opted for single-point urine samples instead of 24-hour urine samples to assess phthalate exposure, a choice that could potentially introduce measurement errors. Replication of these findings is crucial, and further studies are warranted for validation. Secondly, we formed a composite variable by weighting 11 PAEs metabolites to examine the relationship between overall exposure and hormones. At present, we have not explored the influence of each PAEs metabolite on hormones.

In future research, exploring gender and age-specific subgroup analyses could uncover unique patterns in the link between PAEs and obesity, shedding light on different roles within these demographics. Additionally, employing advanced statistical methods, such as deep learning on longitudinal data, may provide a more comprehensive understanding of the potential relationship between PAEs exposure and obesity, capturing the long-term effects of PAEs in the development of obesity. Finally, integrating *in vivo* and *in vitro* methods, such as cell culture experiments or animal models, could contribute to a more in-depth understanding of the biological impact of PAEs on sex hormones and their mechanistic relationship with obesity.

## 5 Conclusions

Our study explores the impact of phthalate exposure on sex steroid hormone levels and the propensity for obesity in adults. The results from the PCA indicate that PC2, primarily composed of MCNP, MCOP, and MCPP, shows a positive association with obesity. Specifically, Estradiol is positively correlated with obesity, whereas TT and SHBG exhibit negative associations. Notably, combined exposure to phthalates and their metabolites leads to an increase in estradiol and SHBG levels, while decreasing TT levels. Among the machine learning algorithms utilized, the RF and XGBoost models demonstrate the highest capability to distinguish adult obesity status. Additionally, the interpretation of both models underscores the effectiveness of sex steroid hormones as predictor variables, highlighting the recommendation to consider them in future obesity-related studies.

## Data availability statement

The original contributions presented in the study are included in the article/**Supplementary Material**. Further inquiries can be directed to the corresponding authors.

## Ethics statement

All NHANES programs were approved by the National Centrefor Health Statistics Ethics Review Committee and written informedconsent was obtained from all participants.

## Author contributions

JZ: Data curation, Validation, Writing – review and editing. WG: Data curation, Formal Analysis, Validation, Visualization, Writing – original draft. SZ: Validation, Writing – review and editing. YL: Validation, Writing – review and editing. CY: Conceptualization, Data curation, Supervision, Writing – original draft. LX: Conceptualization, Data curation, Methodology, Project administration, Supervision, Writing – review and editing. DC: Conceptualization, Supervision, Writing – review and editing.
