# Supplementary material for: Phthalate metabolites and sex steroid hormones in relation to obesity in US adults: NHANES 2013-2016
Source: Front Endocrinol (Lausanne). 2024 Mar 8;15:1340664. doi: 10.3389/fendo.2024.1340664 (PMC10957739; doi:10.3389/fendo.2024.1340664)
Supplement: Supplementary file 1 [file DataSheet_1.docx]

Supplementary materials for

**Phthalate metabolites and sex steroid hormones in relation to obesity in US adults: NHANES 2013-2016**


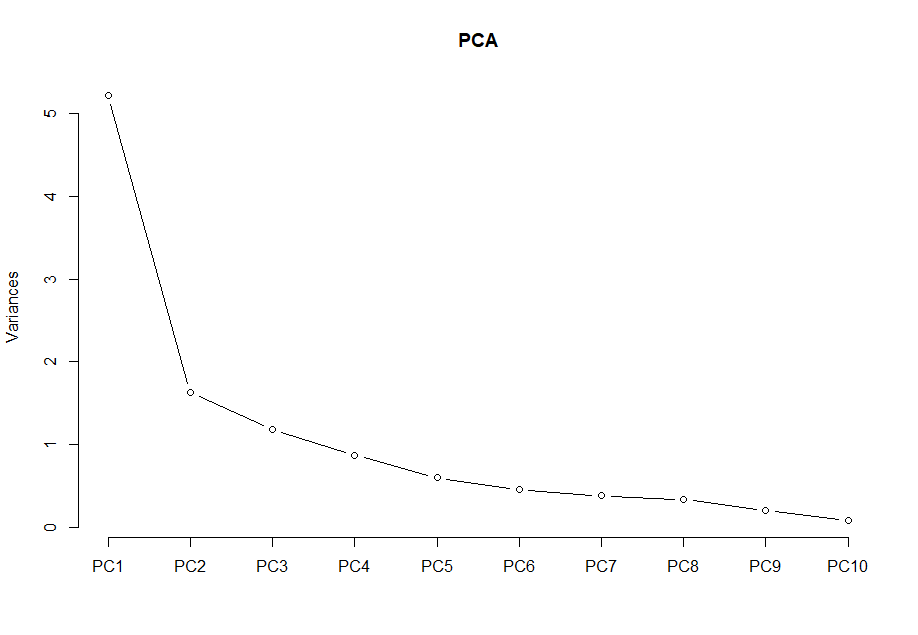


**Figure S1.** The original phthalate metabolite variables were transformed into 11 principal components (PC) and the top six principal components were chosen which explained 90.5% of the total variance of the original variables (The proportion of PC 11 is less than 0.5%).

**Table S1.** Weighted statistical descriptions of the participants.

|  | **All** | **Normal weight** | **Overweight** | **Obese** | ***P* values** |
| --- | --- | --- | --- | --- | --- |
| *n* (%) | 7780 (100) | 1643 (21.12) | 2372 (30.49) | 3765 (48.39) |  |
| *N* (%) | 474487531 (100) | 103244273 (21.76) | 140718303 (29.66) | 230524955 (48.58) |  |
| Age(years); median (IQR) | 57 (25) | 52 (33) | 59 (25) ^a***^ | 57 (22) ^a**^ | 0.002 |
| Gender(male), *N* (%) | 238472329 (50.26) | 40104773 (38.84) | 82838071 (58.87) | 115529486 (50.12) | < 0.001 |
| Race/ethnicity, *N* (%) |  |  |  |  | < 0.001 |
| Mexican American | 33349963 (7.03) | 4213117 (4.08) | 10310704 (77.33) | 18826142 (8.17) |  |
| Non-Hispanic White People | 322825122 (68.04) | 8473230 (8.21) | 13553634 (9.63) | 31621196 (13.72) |  |
| Non-Hispanic Black People | 53648060 (11.31) | 72107202 (69.84) | 96834244 (68.81) | 153883676 (66.75) |  |
| Other Hispanic | 22269917 (4.69) | 3896718 (3.77) | 7556229 (5.37) | 10816970 (4.69) |  |
| Other Race | 42394468 (8.93) | 14554006 (14.10) | 12463492 (8.86) | 15376970 (6.67) |  |
| Education, *N* (%) |  |  |  |  | 0.002 |
| Lower than high school | 80967698 (17.06) | 18051351 (17.48) | 23619062 (16.78) | 39297285 (17.05) |  |
| High school | 103597704 (21.83) | 22003416 (21.31) | 30580541 (21.73) | 51013746 (22.13) |  |
| Some college or AA degree | 158918087 (33.49) | 30303433 (29.35) | 38958763 (27.69) | 89655891 (38.89) |  |
| College graduate or above | 131004043 (27.61) | 32886072 (31.85) | 47559938 (33.80) | 50558033 (21.93) |  |
| Family PIR^1^, *N* (%) |  |  |  |  | 0.017 |
| <= 1.3 | 105594556 (22.25) | 28373341 (27.48) | 26645037 (18.94) | 50576178 (21.94) |  |
| 1.3 ~ 3.5 | 184884967 (38.97) | 39293997 (38.06) | 48892204 (34.74) | 96698765 (41.95) |  |
| > 3.5 | 184008009 (38.78) | 35576935 (34.46) | 65181062 (46.32) | 83250012 (36.11) |  |
| Marital status, *N* (%) |  |  |  |  | < 0.001 |
| Married | 264321034 (55.71) | 45185983 (43.77) | 89849892 (63.85) | 129285158 (56.08) |  |
| Other | 210166498 (44.29) | 58058290 (56.23) | 50868411 (36.15) | 101239797 (43.92) |  |
| Country of birth, *N* (%) |  |  |  |  | < 0.001 |
| US born | 406020764 (85.57) | 83358862 (80.74) | 116967427 (83.12) | 205694475 (89.23) |  |
| Non-US born | 68466768 (14.43) | 19885411 (19.26) | 23750877 (16.88) | 24830480 (10.77) |  |
| [Alcohol](file:///D:\%E7%99%BE%E5%BA%A6%E7%BF%BB%E8%AF%91\baidu-translate-client\resources\app.asar\app.html) use status (yes), *N* (%) | 75882694 (15.99) | 14207091 (13.76) | 17965118 (12.77) | 43710484 (18.96) | 0.013 |
| [Hypertension](file:///D:\%E7%99%BE%E5%BA%A6%E7%BF%BB%E8%AF%91\baidu-translate-client\resources\app.asar\app.html) (yes), *N* (%) | 262803855 (55.39) | 40678320 (39.4) | 68841122 (48.92) | 153284412 (66.49) | < 0.001 |
| Smoking status, *N* (%) |  |  |  |  | 0.090 |
| Current smoker | 92826336 (19.56) | 26045831 (25.23) | 24045507 (17.09) | 42734998 (18.54) |  |
| Former smoker | 142684114 (30.07) | 22456079 (21.75) | 44553318 (31.66) | 75674717 (32.83) |  |
| Never smoker | 238977081 (50.37) | 54742363 (53.02) | 72119478 (51.25) | 112115240 (48.63) |  |
| Physical activity, *N* (%) |  |  |  |  | 0.847 |
| High | 83559600 (17.61) | 17107359 (16.57) | 25842152 (18.36) | 40610089 (17.62) |  |
| Low | 390927932 (82.39) | 86136914 (83.43) | 114876151 (81.64) | 189914866 (82.38) |  |
| Diabetes (yes), *N* (%) | 120164093 (25.33) | 9585649 (9.28) | 26454288 (18.8) | 84124157 (36.49) | < 0.001 |
| Energy intake (kcal/day); median (IQR) | 1994 (791.5) | 1890.5 (772.76) | 2038.97 (775) ^a*^ | 1994 (850.5) | 0.038 |
| Time of blood draw, *N* (%) |  |  |  |  | 0.356 |
| Morning | 241755107 (50.95) | 56738392 (54.96) | 70705243 (50.25) | 114311473 (49.59) |  |
| Afternoon | 168353447 (35.48) | 33433790 (32.38) | 53703350 (38.16) | 81216307 (35.23) |  |
| Evening | 64378977 (13.57) | 13072091 (12.66) | 16309710 (11.59) | 34997175 (15.18) |  |

Sampling weights were applied for calculation of demographic descriptive statistics and N represents the weighted sample size. Mann-Whitney U test and Kruskal-Wallis’ rank sum test were used to compare the differences in continuous variables between subgroups. Chi-square test was used to find differences in categorical variables between subgroups. *P* values for multiple comparisons were calibrated using the Bonferroni’s correction.

a: Compared to the ‘Normal weight’ subgroup.

**P* < 0.05, ***P* < 0.01 and ****P* < 0.001.

^1^ Family PIR represents the ratio of family income to poverty.

**Table S2.** Weighted statistical descriptions of serum sex steroid hormones, urinary creatinine, and urinary phthalate metabolites.

|  | **Overall** | **Normal weight** | **Overweight** | **Obese** | ***P* values** |
| --- | --- | --- | --- | --- | --- |
| *n* (%) | 7780 (100) | 1643 (21.12) | 2372 (30.49) | 3765 (48.39) |  |
| *N* (%) | 474487531 (100) | 103244273 (21.76) | 140718303 (29.66) | 230524955 (48.58) |  |
| Urinary creatinine (mg/dL); median (IQR) | 108 (101) | 92 (102) | 101 (99) | 117 (100) ^a**, b**^ | < 0.001 |
| Phthalates (ng/mL); median (IQR) |  |  |  |  |  |
| MBP | 10.1 (13.8) | 9.7 (14.7) | 9.1 (12.6) | 11 (14) ^b*^ | 0.017 |
| MBzP | 3.6 (6.8) | 3.8 (7) | 3 (6) | 3.8 (7.4) ^b**^ | < 0.001 |
| MCNP | 2 (2.7) | 1.8 (2.6) | 1.9 (2.6) | 2.1 (2.7) ^a**, b*^ | 0.011 |
| MCOP | 9.8 (24.4) | 8.1 (19.2) | 10 (24.8) | 11.5 (28.1) ^a**, b*^ | 0.007 |
| MCPP | 1.3 (2.3) | 1.1 (2.3) | 1.1 (2.2) | 1.4 (2.6) ^b**^ | 0.022 |
| MECPP | 9.4 (12.2) | 8.4 (13.6) | 8.6 (11.1) | 10.2 (12.5) ^b***^ | 0.002 |
| MEHHP | 6.2 (8.6) | 5.5 (8.7) | 5.5 (8.2) | 6.9 (8.4) ^b**^ | 0.014 |
| MEHP | 0.9 (1.53) | 1 (1.83) | 0.57 (1.53) ^a*^ | 0.9 (1.33) | 0.126 |
| MEOHP | 4 (5.5) | 3.6 (5.8) | 3.6 (5.1) | 4.2 (5.7) ^b**^ | 0.013 |
| MEP | 31.4 (84.6) | 25.1 (69) | 23.6 (58.7) | 38.1 (100.6) ^b**^ | 0.007 |
| MiBP | 8.1 (10.8) | 6.6 (10.7) | 7.1 (10.6) | 8.9 (10.9) ^a**, b*^ | 0.003 |
| Serum sex hormones; median (IQR) |  |  |  |  |  |
| Total testosterone (ng/dL) | 129.26 (346.7) | 32.33 (401.2) | 268 (391.6) ^a***^ | 137 (298.9) ^b***^ | < 0.001 |
| Estradiol (pg/mL) | 21.9 (24.3) | 19.9 (34.74) | 22.2 (20.35) | 23.4 (23.2) | 0.075 |
| SHBG (nmol/L) | 52.7 (38.14) | 71.61 (56.44) | 54.95 (36.61) ^a***^ | 46.01 (29.84) ^a***, b***^ | < 0.001 |

Sampling weights were applied for calculation of demographic descriptive statistics and N represents the weighted sample size. Mann-Whitney U test and Kruskal-Wallis’ rank sum test were used to compare the differences between subgroups. *P* values for multiple comparisons were calibrated using the Bonferroni’s correction.

a: Compared to the ‘Normal weight’ subgroup.

b: Compared to the ‘Overweight’ subgroup.

**P* < 0.05, ***P* < 0.01 and ****P* < 0.001.

**Table S3.** Contribution of each phthalate metabolite to each PC.

|  | **PC 1** | **PC 2** | **PC 3** | **PC 4** | **PC 5** | **PC 6** |
| --- | --- | --- | --- | --- | --- | --- |
| MBP | 0.301 | -0.140 | 0.418 | 0.233 | 0.237 | -0.205 |
| MBzP | 0.242 | -0.094 | 0.403 | 0.382 | -0.750 | 0.078 |
| MCNP | 0.255 | 0.455 | 0.002 | 0.003 | 0.042 | 0.814 |
| MCOP | 0.237 | 0.588 | -0.026 | -0.027 | 0.036 | -0.259 |
| MCPP | 0.281 | 0.495 | -0.001 | 0.014 | -0.030 | -0.451 |
| MECPP | 0.390 | -0.131 | -0.251 | -0.054 | -0.056 | -0.011 |
| MEHHP | 0.383 | -0.213 | -0.281 | -0.076 | -0.043 | -0.026 |
| MEHP | 0.324 | -0.206 | -0.274 | -0.070 | 0.065 | 0.070 |
| MEOHP | 0.391 | -0.207 | -0.254 | -0.054 | -0.064 | -0.037 |
| MEP | 0.128 | -0.059 | 0.446 | -0.870 | -0.155 | 0.002 |
| MiBP | 0.277 | -0.151 | 0.427 | 0.159 | 0.583 | 0.104 |

The top six principal components were chosen which explained 90.5% of the total variance of the original variables.

PC: Principal component.

**Table S4.** Phthalate metabolites.

| **Abbreviations** | **Metabolite** | **LLOD (ng/mL)^1^** |
| --- | --- | --- |
| MBP | mono-n-butyl phthalate | 0.4 |
| MBzP | monobenzyl phthalate | 0.3 |
| MCNP | monocarboxyononyl phthalate | 0.2 |
| MCOP | monocarboxyoctyl phthalate | 0.3 |
| MCPP | mono(3-carboxypropyl) phthalate | 0.4 |
| MECPP | mono(2-ethyl-5-carboxypenty) phthalate | 0.4 |
| MEHHP | mono(2-ethyl-5-hydroxyhexyl) phthalate | 0.4 |
| MEHP | mono(2-ethylhexyl) phthalate | 0.8 |
| MEOHP | mono(2-ethyl-5-oxohexyl) phthalate | 0.2 |
| MEP | mono-ethyl phthalate | 1.2 |
| MiBP | mono-isobutyl phthalate | 0.9 |

^1^ LLOD, lower limit of detection

**Table S5.** Multinomial logistic regression results for sub-analyses.

|  | **Middle-aged** | | **Older** | |
| --- | --- | --- | --- | --- |
|  | **Male** | **Female** | **Male** | **Female** |
| **Sex steroid hormone** |  |  |  |  |
| TT | **0.996 (0.995, 0.997)** | **1.017 (1.008, 1.027)** | **0.989 (0.987, 0.990)** | 1.000 (0.992, 1.008) |
| Estradiol | **1.014 (1.001, 1.027)** | 0.998 (0.995, 1.002) | **1.244 (1.214, 1.274)** | **1.065 (1.036, 1.077)** |
| SHBG | **0.966 (0.956, 0.976)** | **0.980 (0.977, 0.983)** | **0.992 (0.985, 0.999)** | **0.966 (0.961, 0.970)** |
| **Principal components of PAEs ^1^** |  |  |  |  |
| PC 1 | 0.917 (0.838, 1.002) | 0.958 (0.885, 1.038) | **0.704 (0.635, 0.781)** | **0.810 (0.741, 0.885)** |
| PC 2 | **1.285 (1.147, 1.440)** | **1.119 (1.020, 1.227)** | 0.991 (0.861, 1.142) | 1.019 (0.886, 1.172) |
| PC 3 | 0.900 (0.779, 1.041) | **1.241 (1.086, 1.418)** | **0.798 (0.692, 0.920)** | **1.291 (1.109, 1.504)** |
| PC 4 | 0.970 (0.821, 1.147) | 1.155 (0.993, 1.343) | **1.504 (1.283, 1.763)** | **0.772 (0.649, 0.918)** |
| PC 5 | 1.042 (0.878, 1.237) | 0.950 (0.801, 1.128) | 0.935 (0.788, 1.110) | 1.004 (0.826, 1.221) |
| PC 6 | 1.007 (0.913, 1.110) | 1.187 (0.985, 1.430) | **1.194 (1.085, 1.314)** | **0.539 (0.438, 0.663)** |

The results of the multinomial logistic regression are expressed as ORs and 95% CIs, with ‘Obese’ compared to ‘Normal weight’. Bolded ORs indicate statistical significance (p<0.05). The proportion of each phthalate metabolite in each PC is shown in Table S3. Participants were categorized as ‘middle-aged’ and ‘older’ based on median age (60).

^1^ Principal components consisting of the original PAEs variables.

TT: Total testosterone; SHBG: Sex hormone-binding globulin; OR: Odds ratios; CI: Confidence intervals; PC: principal component; PAEs: phthalates.

**Table S6.** WQS regression results for sub-analyses.

| **PAEs indexes of sub-analyses** | **Total testosterone ^1^** | **Estradiol ^2^** | **SHBG ^3^** | **BMI ^4^** |
| --- | --- | --- | --- | --- |
| Middle-aged | **-112.00 (-134.74, -89.26)** | **3.95 (0.43, 7.47)** | **11.50 (8.13, 14.87)** | **0.73 (0.02, 1.45)** |
| Older | **-54.70 (-78.22, -31.18)** | **2.82 (1.01, 4.62)** | **3.20 (0.19, 6.21)** | **0.72 (0.11, 1.33)** |
| Male-mid | **-58.48 (-88.29, -28.66)** | **-8.81 (-10.89, -6.74)** | 1.19 (-1.54, 3.92) | **1.49 (0.79, 2.19)** |
| Female-mid | **-8.99 (-12.74, -5.24)** | 4.32 (-1.19, 9.83) | 2.12 (-4.05, 8.28) | **1.17 (0.16, 2.19)** |
| Male-old | **-72.67 (-109.73, -35.60)** | **4.93 (2.98, 6.87)** | **8.74 (4.54, 12.93)** | **1.23 (0.36, 2.10)** |
| Female-old | **-11.88 (-15.46, -8.31)** | **1.37 (0.24, 2.50)** | **5.60 (0.33, 10.87)** | **3.04 (2.00, 4.09)** |

Regression coefficients and 95% CIs for mixed exposures in the weighted quantile sum regression. Bolded coefficients indicate statistical significance (*P*<0.05). Participants were categorized as ‘middle-aged’ and ‘older’ based on median age (60).

^1^ WQS regression model with total testosterone as the dependent variable.

^2^ WQS regression model with estradiol as the dependent variable.

^3^ WQS regression model with SHBG as the dependent variable.

^4^ WQS regression model with continuous BMI as the dependent variable.

TT: Total testosterone; SHBG: Sex hormone-binding globulin; PAEs: phthalates; CI: Confidence intervals; WQS: Weighted quantile sum; BMI: Body mass index.
